# Retraction: Upregulation of microRNA-497-5p inhibits colorectal cancer cell proliferation and invasion via targeting PTPN3

**DOI:** 10.1042/BSR-20191123_RET

**Published:** 2020-08-03

**Keywords:** Colorectal cancer (CRC), invasion, microRNA-497-5p (miR-497-5p), proliferation, protein tyrosine phosphatase non-receptor type 3 (PTPN3)

This Retraction follows an Expression of Concern relating to this article previously published by Portland Press.

This article is being retracted from *Bioscience Reports* at the request of the Editor in Chief and Editorial Board following receipt of a notification from several readers, alerting the Editorial Office to manipulation in Figures 1B, 2C, 4C, and 4D.

The authors have been contacted in regard to the retraction, and have not responded to the Journal. Given the extent of the issues raised, Editorial Board stand by the decision to retract the article.

